# Indications and technical aspects of proximal gastrectomy

**DOI:** 10.3389/fsurg.2023.1115139

**Published:** 2023-02-16

**Authors:** Peter Kolozsi, Zsolt Varga, Dezso Toth

**Affiliations:** Department of Surgery, University of Debrecen, Debrecen, Hungary

**Keywords:** gastric cancer, proximal gastrectomy, minimally invasive surgeries, laparoscopic gastrectomy, upper third gastric cancer, early gastric cancer

## Abstract

According to the World Health Organization, gastric cancer is the fifth most common type of tumor, and is the third most common cause of tumor-associated death. Although gastric cancer incidence rates have decreased in the past few decades, the prevalence of proximal gastric cancer has been steadily rising in developed countries. Techniques regarding the improvement of treatment options must thus be developed. This can be achieved through incorporating both a wider use of endoscopic surgery (endoscopic mucosal resection—EMR, endoscopic submucosal dissection—ESD) and a review of applied surgical interventions. Even though there is no single international consensus available, the Japanese Gastric Cancer Association (JGCA) recommends proximal gastrectomy with D1+ lymphadenectomy in early gastric tumors. Despite recommendations from Asian guidelines and the short term outcomes of the KLASS 05 trial, surgical treatments in Western countries still rely on total gastrectomy. This is mostly due to technical and oncological challenges regarding surgical interventions in a proximal gastrectomy. However, the residual stomach after a proximal gastrectomy has been shown to diminish the incidence of dumping syndrome and anemia, and even improve postoperative quality of life (QoL). Therefore, it is necessary to define the place of proximal gastrectomy in the treatment of gastric cancers.

## Introduction

Incidence rates of gastric cancer have changed significantly in the past decades: in 2008, gastric carcinoma was considered the fourth most common malignancy, and the second most common cause of cancer-associated death; its incidence significantly declined by 2020 (1). According to WHO data, gastric carcinoma is currently the fifth most common type of tumor. It is also the third most common cause of tumor-associated mortality following lung and colorectal cancer (2). This decline in mortality may be due to systematic exploration of various risk factors, such as the leading role of Helicobacter pylori, an eradicable pathogen (3), as well as lifestyle factors promoting the incidence of gastric cancer, including high salt intake, smoking, or alcohol abuse (4, 5). Early endoscopic diagnosis, perioperative oncological treatment and surgical care must therefore be incorporated. Nevertheless, the overall five-year survival rate of gastric cancer in Western societies is still as low as 20% due to frequently late diagnosis. In comparison, Asian countries (South Korea, Japan) run complex screening programs for upper gastrointestinal cancer, and tend to diagnose gastric carcinomas at an early stage; their five-year survival rate there is nearly 70% for stage I and II (6).

Furthermore, even though the overall incidence of gastric cancer is decreasing, proximal gastric carcinoma cases are still on the rise (7). This increased incidence and subsequent decline in quality-of-life indices applied after a “gold standard” total gastrectomy (TG) has called for a paradigm shift in therapy. This can be reflected by the increasing application of endoscopic resection methods (endoscopic mucosal resection [EMR] and endoscopic submucosal dissection [ESD]). As a result of ESD allowing wider *en bloc* resection (presented first by Gotoda et al. in 1999), these techniques cover more than 60% of all procedures in Japan for the treatment of early gastric cancers (EGC) (8–10). In cases where endoscopic methods are not feasible, proximal gastrectomy (PG) may be a reasonable alternative for TG. This is due to its shorter operation time, lower intraoperative blood loss and the better nutritional status in the postoperative period of patients who underwent PG. Even though there is an increasing amount of data on the oncological safety and technical feasibility of proximal gastrectomy, there is no international consensus providing a standardized guideline for the operative therapy in upper third gastric tumors. This is well shown by that their number is rather low regarding the Eastern countries most supporting PG, contrary to the changes in the incidence indices of gastric tumors. In South Korea, in 2009, these types of surgeries represented only 1% of all gastric tumor related surgical interventions, including open and laparoscopic surgeries (11). While in 2013 in Japan the number of proximal laparoscopic resections was as high as 4.6%, which was at the time, higher than the number of the open PG (12). Their increasing trend in the previous decade was constant mostly due to the Asian countries. The purpose of this review is to summarize the current status of PG in gastric surgery. Our aim is review PG's oncological radicality and discuss the important aspects of indication and technical applicability. Furthermore, the reconstructive procedures following PG that greatly influence postoperative short- and long-term results, will be presented in detail.

## Technical aspects of PG

### Oncological safety of PG

The use of oncological radicality in distal laparoscopic gastrectomy for distal gastric carcinomas is currently standard practice (11–13). The first line treatment for advanced upper third proximal gastric cancer, however, is still TG with D2 lymphadenectomy (14). The treatment method of early proximal gastric cancer underwent significant changes in the past decades. In early gastric carcinoma cases where endoscopic methods are unnecessary, a proximal gastrectomy may be performed as a suitable alternative to TG. Regardless, the basic surgical treatment of early upper third gastric tumors in Western countries is identical to that of advanced tumors. In Asian countries, developed complex care includes screening programs ensuring early diagnostics, gastroenterological interventions, such as the ones detailed above, and cutting-edge minimally invasive surgical techniques. This has resulted in improvement of the well-registered survival indices which brought both oncological results and postoperative quality of life into focus. Owing to the above, certain subtypes of PG presumably providing functional benefits in terms of nutrition, emerged.

The oncological radicality of proximal gastrectomy in the treatment of early gastric cancers has been questioned by surgeons. The extension of surgical procedures—in addition to defining the place of PG—is also a controversial, for example indication for complete omentectomy vs. partial omentectomy, given the fact that the incidence of omental metastases in T3–T4 gastric cancer is only 3.8%–5% (15). Other doubts regarding oncological radicality have now been resolved, including the use of laparoscopy in early and advanced gastric cancer (CLAAS – 01 trial) (16), the estimation of probability of lymph node metastasis using the Maruyama computer program (17) and the extension of lymph node dissection performed in advanced gastric cancer.

The indication of PG is currently for early upper-third gastric cancers, but the latest studies are increasingly pointing beyond this, even for locally advanced cases. A study by Yura et al. reported that advanced (T2–T3) gastric tumors located in the upper third of the stomach had relatively low metastasis rates in the infra- or suprapyloric lymph nodes. In quantitative terms: their data analysis for both T2 and T3 gastric tumors showed a 0% rate for lymph node metastatic potential in stations 5 and 6 (18). A study by Ri et al. showed that locally advanced T2–T4 gastric tumors at the level of the cardia and fornix did not show metastatic potential in the lymph node stations 4, 5, 6, and 12a. In these cases, a PG is permissible. At the same time, tumors that infiltrated the gastric body showed an increased possibility of metastasis in the distal lymph nodes. Accordingly, the role of PG in the treatment of these tumors is highly questionable (19). Similar to Yura's and Ri's data, Takeuchi et al. did not find metastasis in the lymph nodes 5, 6, 10 or 11d in early upper third (T1N0) gastric tumors either (20). A similar conclusion was also reached i.e., lymph node station 5 and 6 had a metastatic potential of 0.5% and 1.6%, respectively. With the notion of PG oncological radicality in mind, Haruta's study group found that all tumors in the upper third of the stomach that measured less than 4 cm, whose distal border also ended in the upper third, had low (2.2%, *p* < 0.001) rates of 3b lymph node metastasis (3b lymph nodes: distal lymph nodes of the lesser curvature, located along the right gastric artery), thus 3b lymphadenectomy was not necessary (21). This conclusion was further supported by sentinel lymph node (SLN) mapping of tumors in the upper third of the stomach, which were identified using double-guided (radio- and dye-guided) methods. According to Niihara, the incidence rate of parapyloric presentation of SLN from these tumors is around 0%–3%, and zero at station 8. Therefore, PG excluding the dissection of these lymph node stations can be performed with oncological safety (22).

As defined by the JGCA, upper gastric tumors are located in the upper third of the stomach, with or without the involvement of the esophagogastric junction (EGJ). EGJ tumors, however, should be mentioned as a specific indication for PG. Yamashita et al. found that the metastatic potential of EGJ tumors below 4 cm in lymph node stations No. 1, 2, 3, and 7 was particularly high, even in esophageal-predominant tumors. The susceptibility for metastasis in lymph node stations No. 4, 5, and 6 was almost zero, regardless of the esophageal or gastric predominance of the EGJ tumor or the T stage. Thus, for EGJ tumors less than 4 cm, removal of distal lymph nodes around the stomach is not indicated (23). This was also supported by a meta-analysis by Li et al., who concluded that PG may be the most appropriate procedure for Siewert II-III. tumors, considering both the oncological radicality and postoperative functional benefits (24). Kurokawa et al. conducted a prospective nationwide study in collaboration with the JGCA and the Japanese Esophageal Society (JES). They reported that performing a distal esophagectomy combined with PG is sufficient for Siewert II. EGJ tumors, regardless of the presence of adenocarcinoma or squamous cell carcinoma. A total gastrectomy and paraaortic lymph node dissection (LND) is not necessary; however, mediastinal lymphadenectomy should be considered for esophageal involvement (EI) above 2 cm. (EI > 2 cm – 110 LND; EI > 4 cm – 106 right recurrent laryngeal nerve LND) (25).

With regards to long-term oncological outcomes, it is worth analyzing the rate of local recurrence. The 2004 study by Yoo et al. introduced findings of a former period of proximal gastric tumor surgeries. By processing data from 74 patients who had undergone a PG, and 185 patients who had undergone a TG, they experienced that out of 66 patients to PG (8 patients had R1 resection) local recurrence appeared for 17 (25.7%). The authors explained this high rate by the more extended or so to say less-defined circle of indication, including the selected malignancies with serosal infiltration (T4 tumors), the diffuse tumor type or the tumor size over 5 cm (26). Similar recurrence rates were also found for proximal and total gastrectomy's performed in patients with stage IA and IB gastric tumors (below 4 cm in size, located in the upper third). The same was found by Chen et al. in their meta-analysis where the five-year overall survival rate [odds ratio (OR): 0.95, 95% CI, 0.64–1.40; *p* = 0.790] and recurrence ratio (OR: 3.79, 95% CI, 0.37–38.46; *p* = 0.260) of proximal and total gastrectomy's were similar (27). The systematic review and meta-analysis performed by Xu et al. concluded the same upon comparing the two types of surgery (OR: 0.841, 95% CI, 0.549–1.287 *p* = 0.430) (28), which was seen in the level of significance as well owing to Li's analysis having processed the data of 1,734 patients in 12 studies (OR: 1.35, 95% CI, 0.99–1.85, *p* = 0.06) (24).

### Feasibility of PG

Regarding technical feasibility, several aspects must be considered. First, whether laparoscopy used in distal gastrectomy (13) provides advantage in proximal surgeries as well. The first laparoscopically assisted PG was described more than 20 years ago (Kitano et al., 1999) (29). Several retrospective studies were dealing with the benefits of laparoscopic PG, including the well-known: less pain due to minimal invasiveness, faster recovery and easier mobilization of the patient.

**Figure 1:**
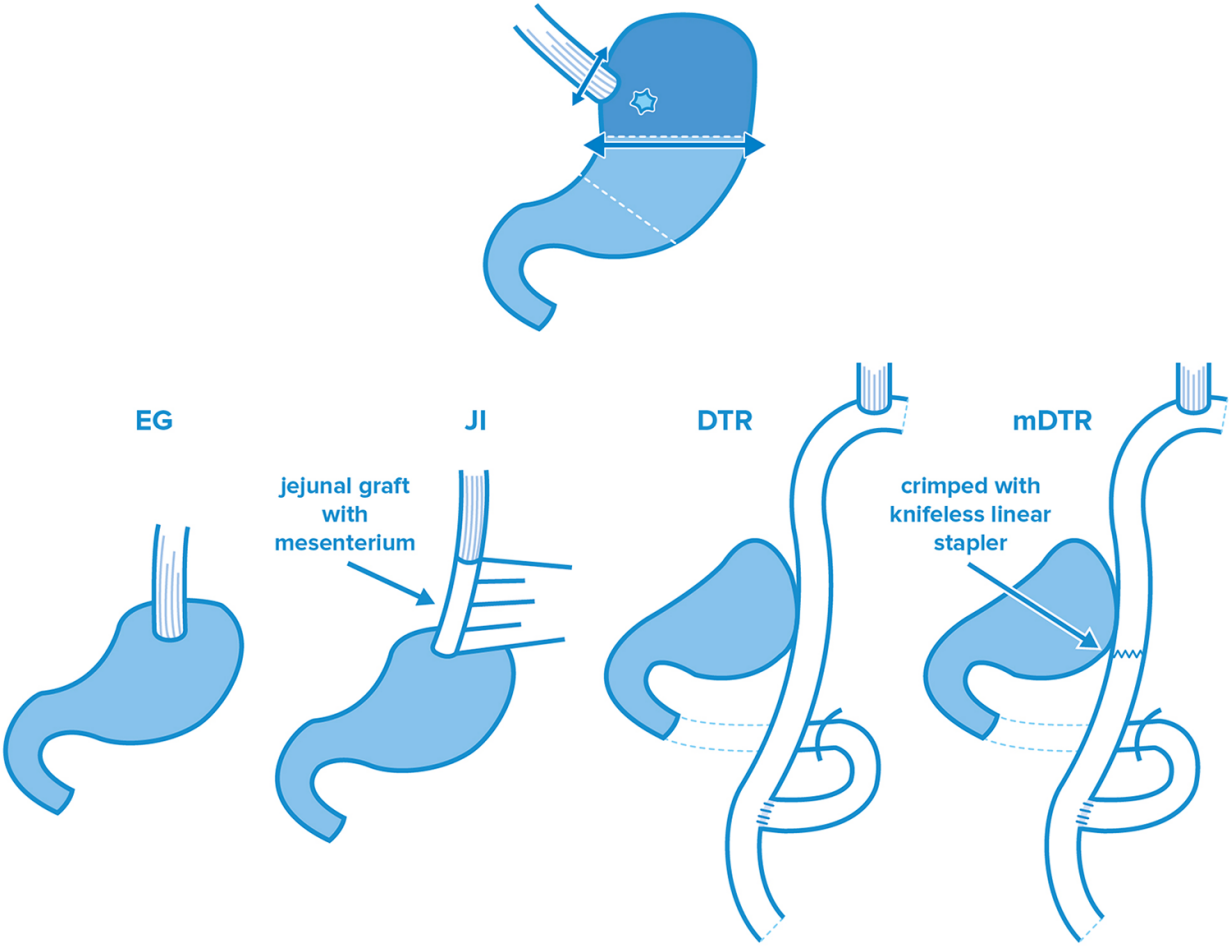
Standard reconstruction procedures after PG: esophagogastric anastomosis (EG), jejunal-interposition technique (JI), double—tract (DTR) and modified double—tract (mDTR) reconstruction.

After PG, there are three standard reconstruction procedures: esophagogastric anastomosis (EG), double—tract reconstruction (DTR) and the jejunal—interposition (JI) technique (Figure 1). However, considering the difficulty of these reconstruction techniques following PG and the subsequent outcomes, laparoscopy was not clearly defined as standard surgical procedure for proximal surgeries. The retrospective analysis performed by Kinoshita et al. compared PGs reconstructed by open and laparoscopic JI. There was no reported difference in lymph node resection, esophagojejunal anastomotic insufficiency or occurrence of postoperative complications. Although the duration of the surgery was significantly longer in the laparoscopic group (233 vs. 201 min., *p* = 0.0002), decreased blood loss (20 vs. 242 grams, *p* = 0.0001) and the reduced need for painkillers after surgery (the number of times of additional analgesia, 2 vs. 4, *p* = 0.0001) were also significant (30). The aim of the JCOG 1,401 single-arm confirmatory trial published in 2019 was to revolutionize retrospective processing and prove the safety of laparoscopically assisted total and proximal gastrectomies with double—tract reconstruction or jejunal—interposition in case of stage I (T1N0, T1N1, T2N0) upper third early-stage gastric tumors. Even though the total esophagojejunal anastomotic insufficiency rate was predicted to be 8% (one-sided *p* = 0.0002), the research showed that patients had a rate of only 2.5%. This insufficiency did not show any difference between the two surgical types (6 cases out of 244 surgeries, 95% CI, 0.9–5.3). Major complications and conversions occurred at a similarly low ratio, and postoperative mortality was found to be zero. Accordingly, the standard surgical intervention recommended by the authors in case of early, stage I proximal gastric malignancies is laparoscopic surgery. However, it should be noted that the authors mentioned these surgeries must be performed only in high-volume centers by accredited upper GI surgeons (31). Similar intraoperative and postoperative aspects can be considered when comparing the findings of laparoscopic total gastrectomy (LTG) with laparoscopic proximal gastrectomy (LPG), According to the findings of the meta-analysis performed by Chen et al. mentioned above, proximal surgeries involve less lymph node removal [weighted mean difference (WMD): −12.86, 95% CI, −17.44 to −8.28; *p* = 0.000] and lower blood loss in the case of LPG (WMD: −102.18, 95% CI, −180.41 to −23.94; *p* = 0.010). However, they require more time (WMD: −65.47, 95% CI, −103.39 to −27.55, *p* = 0.001) and are accompanied by higher rates of anastomotic stenosis (OR: 3.18; 95% CI, 1.46–6.92; *p* = 0.004), the latter of which shows high variability among reconstruction types. The most frequent is in direct EG anastomoses. and the probability of postoperative ileus is also lower than in LTG (OR: 0.27; 95% CI, 0.10–0.72; *p* = 0.010) (27). The meta-analysis performed by Li found a similar relationship regarding intraoperative blood loss and the duration of surgery, and also mentions the better postoperative nutrition level in PG (24).

### Functional benefit of PG

The partial preservation of the reserve function of stomach plays an unambiguous role in the above, which is positively reflected by the postoperative nutrition, the formation and severity of the incidental dumping syndrome and the abundance of postoperative diarrhea episodes. To quantify the above, Ahn et al. found that 6 months after a PG surgery, the loss of body weight was 5.9% compared to a weight loss of 16% found after TG (32). Takiguchi et al. found similar outcomes in their study, with patients experiencing significantly higher weight loss after TG (TG 13.8% vs. PG 10.9%, *p* = 0.003) (33). Weight loss alone is not sufficient enough to assess postoperative status, so analysis of qualitative indices must be performed as well. This is achieved through the monitoring of serum hemoglobin, serum albumin, total protein and Vitamin B12 level. Some studies did not find any significant difference between the two types of surgery in terms of nutritive findings. In their multicentric, prospective and non-randomized study, Yamasaki et al. saw no significant difference in serum albumin and hemoglobin levels in the short term postoperative period. There was also no significant difference in Vitamin B12 levels one year after surgery (4.2% vs. 7.2%, *p* = 0.07), however there was a significant difference after the two year mark (2.2% vs. 7.7%, *p* = 0.003) (34). Masuzawa experienced similar results regarding the postoperative monitoring of patients, by comparing PGs reconstructed by EG JI to those operated on using the Roux-en-Y TG technique. 3 years after the surgery, the patients subject to TG had lower serum levels of both albumin and hemoglobin (albumin *p* = 0.012, hemoglobin *p* = 0.046) (35), showing the advantage of PG's in long-term nutritive status. However, functional advantages after PG occur only if at least half of the stomach remains after PG resection. Accordingly, based on the most recent Japanese Gastric Cancer Treatment Guideline, 2021, PG is only recommended if at least 50% of the whole volume of the stomach is retained (36).

We currently have high-quality randomized prospective research on retrospective processing, owing to the KLASS – 05 trial. The KLASS – 05 trial is the first randomized multicentric study comparing proximal PG for upper third T1 stage early gastric tumors (by double-tract reconstruction) with TG. The research conducted between October 2016 and September 2018 selected 68 undergoing a PG and 69 TG patients, in order to compare the short-term and long-term effects of these two types of surgery. By analyzing the perioperative stage (by registering the preoperative data and the postoperative data on days two and five) it was concluded that no significant difference was found between proximal and total gastrectomy in terms of certain serum parameters (hemoglobin, albumin, white blood cells, C reactive protein) and other short-term mortality and morbidity indices. However, the authors wrote that PG can still be an alternative to TG for these patients, by considering that the long-term findings are still to come (37).

## Reconstruction types after PG

As mentioned above, the JGCS guideline 6th edition differentiates 3 basic reconstruction procedures after proximal gastrectomy (36): (a) esophagogastric (EG) anastomosis, (b) double—tract technique (DTR) and (c) jejunal—interposition (JI) reconstruction. Although several modified procedures (e.g., gastric tube EG, modified DTR) were elaborated on in the previous decades, no clear recommendation was given for any of these surgeries. Within the framework of this review article, in addition to the governing techniques, we intend to introduce other procedures besides the default which have the potential to even become the new “gold standard” surgery. Accordingly, in addition to the 3 standard surgical reconstruction techniques, the surgeries based on the flap technique aiming at the formation of a new EG sphincter (double—flap technique [DFT], side—overlap fundoplication [SOFY], modified side—overlap funduplication [mSOFY]) are introduced as separate techniques. Both DFT and SOFY are considered a subtype of EG, which try to combine the simplicity of EG anastomosis with the outstanding functional results of the other reconstruction procedures.

### Esophagogastrostomy

In EG, after the removal of the proximal part of the stomach, the restoration between the esophagus and the gastric stump is performed by a circular stapler, predominantly with transorally-inserted anvil (Orvil^™^). Due to the application of JI or DTR, the EG anastomosis type widely applied earlier has somewhat declined. This decline is due to controversy surrounding the high reflux esophagitis to anastomosis stenosis ratio in the postoperative stage (33, 38). However, there is significant variability in terms of the applied surgical technique. Compared to the traditional circular stapler technique, the first promising EG modification linked to Adachi who, in 1999, elaborated the gastric tube EG technique to terminate reflux complaints (39). The basis of this surgery is to provide a significantly longer piper gastric stump to create a greater distance for the bile to travel for reflux.The modification provided by Adachi had outstanding results in terms of reflux esophagitis, however, multiple studies reported higher rate of anastomotic stenosis compared even to the traditional EG surgeries. This surgical technique has thus not been widely accepted. In their retrospective data processing, Ahn et al. found that there is a significant difference between the findings of end-to-end esophagogastrostomy (EEEG) performed by a circular stapler, as compared to side-to-side esophagogastrostomy (SSEG) performed by a linear stapler. After EEEG, stenosis appeared with a rate of 46.2%, a significant difference when compared with the 0% found after SSEG (*p* < 0.001). No significant difference was found in terms of reflux esophagitis during the primary processing (15.4% vs. 37.8%, *p* = 0.135) until the five years of the research was divided into three separate phases (early, middle and late phases) In the late phase, every patient was subject to supplementary esophagopexy by hiatus reconstruction, demonstrating no significant change in reflux symptoms. The late phase results showed no patients with Visick grade IV reflux esophagitis proving that SSEG anastomosis by linear stapler, with supplementary anti-reflux treatment, is a suitable alternative for optimal reconstruction surgery (40).

### Esophagogastrostomy flap techniques: DFT, SOFY

**Figure 2:**
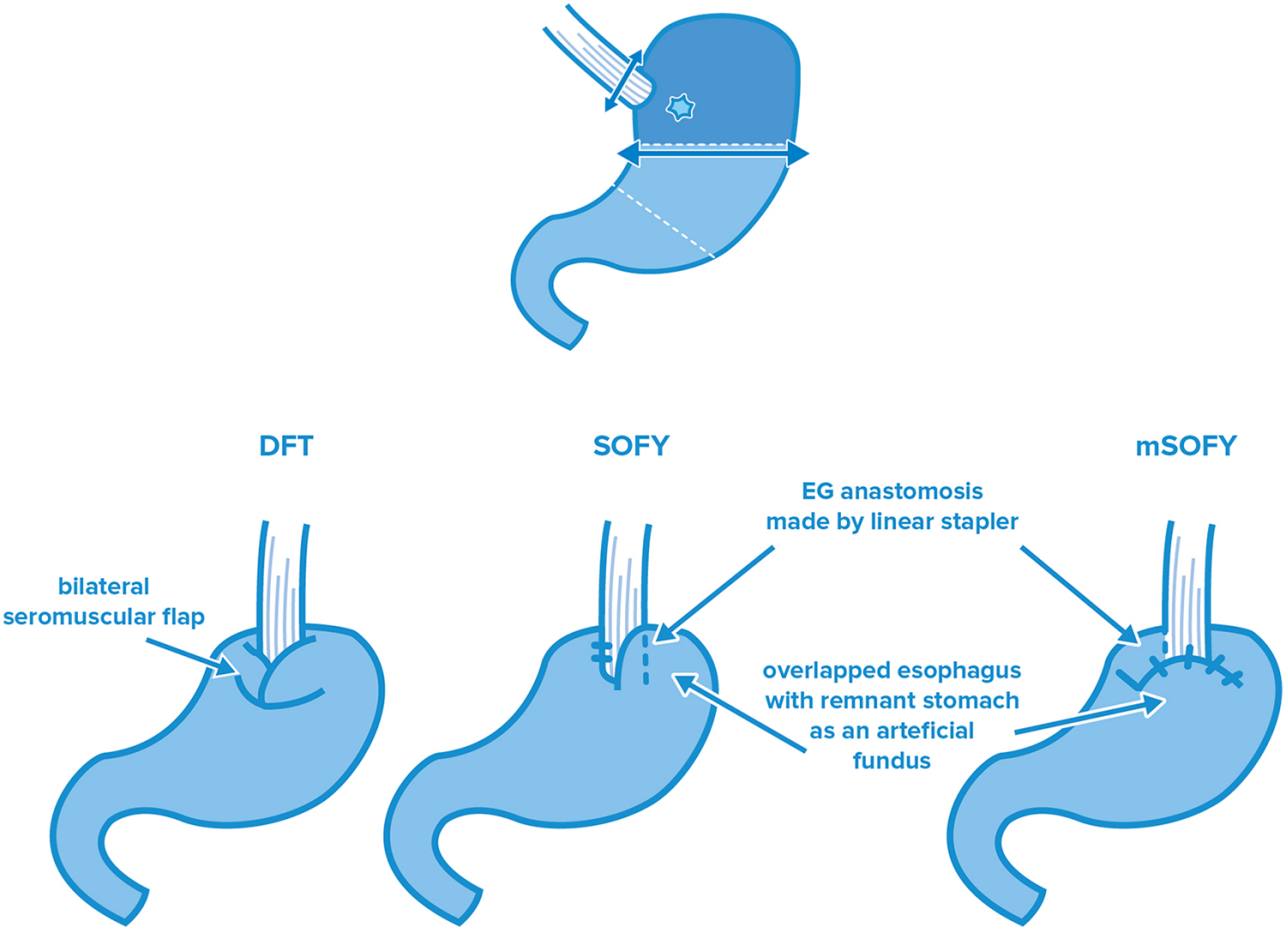
Esophagogastrostomy flap techniques after PG: double—flap technique (DFT), side overlap funduplication (SOFY), and modified side overlap funduplication (mSOFY).

To eliminate reflux complications resulting from traditional EG surgeries, in 2001, Kamikawa et al. devised an anti-reflux technique (41) (Figure 2). The technique involved a hand-sewn laparoscopic EG after the removal of the proximal part of the stomach, which is covered by a sero-muscular flap from two sides. Due to the difficulty of the handmade sutures, this surgical technique was subject to heavy criticism, however the comparative study conducted by Hayami et al. showed outstanding results. They compared the findings of DFT with laparoscopic TG and found that even though DFT requires longer surgery time, it is more favorable in terms of hospitalization time (*p* = 0.002) and the nutritive status of the patients (weight *p* = 0.003, total protein *p* < 0.001, albumin *p* = 0.06, hemoglobin *p* = 0.003) (42). Saze et al. found better outcomes not only compared to the total removal of the stomach by analyzing the change of postoperative weight loss but also after comparing it with certain PG subtypes (traditional EG, JI, DTR, *p* = 0.001–0.013) (43). On the contrary, several studies pointed out that in addition to the technical difficulty and high skill required to perform a DFT, there was also increased risk for stenosis or incidental ischemia following formation of the flap with consequential necrosis (44). To exclude the above, the modified laparoscopic Kamikawa anastomosis was elaborated by Mo et al.; during this, the esophagogastric anastomosis is made by a traditional circular stapler, however, the suture line was covered by a unilateral seromuscular flap. The theoretical basis of this technique is the higher speed of the standardized machine-made anastomosis, the lower stenosis rate compared to the manual suture line and the valve function of the seromuscular flap forming (45).

In side overlap funduplication surgery, the linear stapler EG technique originally performed by Ahn is combined with the sphincter function of the flap. This surgery type was described by Yamashita et al. in 2017, who further modified it in 2022 [modified SOFY (mSOFY)]. In the original technique, a 5 cm overlap between the distal part of the esophagus and the gastric stump is created and anastomosis is formed by turning the linear stapler device counterclockwise and sewing the residual stomach to the left side of the esophagus. In the 2022 modification, anastomosis is formed according to the same method on the right side of the esophagus, which is followed by the formation of a “plica” from the gastric stump, which covers the last part of the esophagus. Owing to this surgical modification (whether it is the original or the modified SOFY procedure), the distal part of the esophagus serves as a neosphincter with a “flat” form. Yamashita et al. found no suture insufficiency for the SOFY nor mSOFY. Anastomosis stricture appeared only once, and outstanding results were obtained in terms of reflux symptoms as well (46, 47). The judgement of the flap-type EG surgeries is complicated by the lack of detailed and prospective data covering a wide period of time both for the modified Kamikawa anastomosis and the SOFY technique. Accordingly, even though their application as standard reconstruction procedure is currently out of question, the monitoring of these surgery types is by all means recommended due to their “simplicity” and efficiency compared to the laparoscopic hand-sewn EG anastomosis.

### Reconstruction with small intestine: double-tract reconstruction (DTR and mDTR), jejunal pouch (JP) and jejunal interposition (JI)

**Figure 3:**
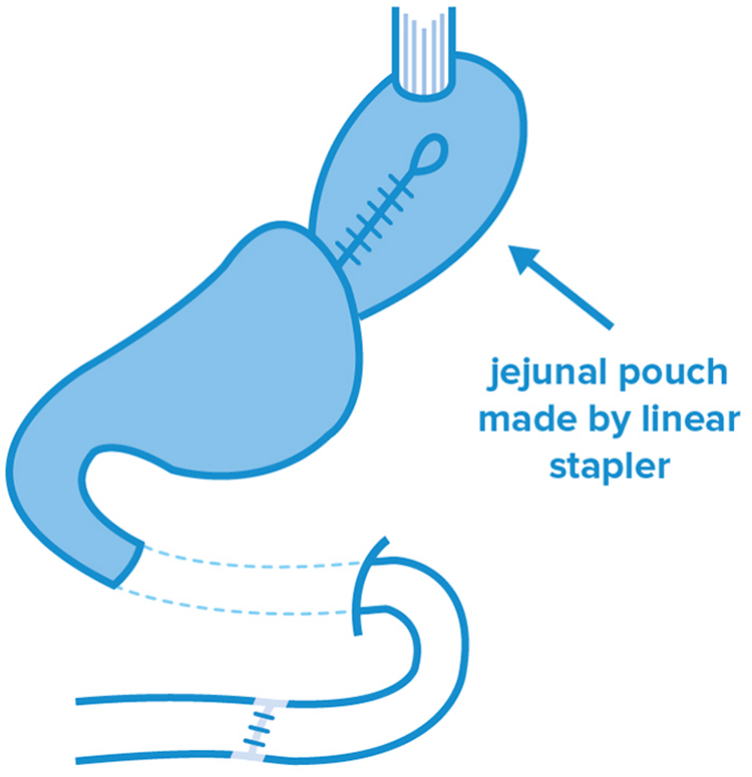
Jejunal pouch technique after PG.

During jejunal reconstruction surgeries, PG is performed by a jejunum loop to restore gastrointestinal continuity. In case of the jejunal pouch technique, a reservoir is formed from an isolated jejunum limb, which is followed by the PG reconstruction stage (Figure 3). In the comparative pilot study performed by Takagawa in 2010, better results were experienced than in case of JI in several aspects. The most striking aspect was the worse short-term morbidity data of JI, including anastomosis insufficiency and postoperative bleeding, surgical site infection (SSI) and postoperative pneumonia (*p* = 0.036) (48). Similarly, better results were experienced after JP in terms of postoperative body weight (*p* = 0.095) and food intake (*p* = 0.002). Although the formation of JP provides a clear advantage in terms of food intake right after surgery, this technique was recently dismissed due to challenges in pouch formation and the abundance of residual food, which was experienced in an extreme extent, for more than 90% of the patients (49).

In case of both DTR and JI surgeries, the dissection plane of the jejunum is set approximately 20 cm from the ligament of Treitz. After the dissection of the jejunum, esophagojejunal (EJ) and gastrojejunal (GJ) anastomoses are formed with the aboral gut section. In order to ensure bile discharge, a jejunal junction (JJ) is formed. To prevent reflux esophagitis, most the authors recommend a distance of 10–15 cm between EJ and GJ anastomoses. In case of DTR, the consumed food is passed on towards the stomach and the jejunum as well; according to the passage study of Ahn, the passage of food is distributed between the residual stomach and the jejunal loop in a ratio of 3 : 2 (32). In case of the modified DTR (mDTR) also known as single-tract jejunal interposition (STJI), the jejunum is closed below the GJ anastomosis by a knifeless linear stapler, which facilitates the passage of the consumed food towards the stomach. The DTR and the STJI techniques have clear advantages when compared to traditional EG anastomoses in terms of reflux esophagitis and anastomotic stenosis (34). Compared to gastrectomy, both techniques are more effective in terms of postoperative nutritive status (35). In the prospective study by Nomura et al. comparing the findings of DTR and STJI, even though no substantial difference was found in the meal intake ratio (postoperative—preoperative meal intake ratio: the mean of the whole postoperative meal intake per day compared to the preoperative meal intake), STJI had significantly better results in terms of postoperative body weight and postprandial serum insulin levels (*p* < 0.05) (50). Lu et al. obtained similar findings: although STJI had significantly longer duration (*p* = 0.04), it proved to be significantly better in terms of postoperative body weight (*p* = 0.002) (51). Accordingly, these work-groups recommended STJI regarding reconstruction following PG surgeries. In the meta-analysis by Wang et al., it was found that early complications, stenosis, reflux esophagitis and residual food appeared at a ratio of 18.1%, 9.6%, 4.5% and 19.0% for JI, and at a ratio of 11.6%, 4.7%, 4.7% and 48.9% for DTR, respectively (52). Most of the authors agree that the higher incidence ratio of early complications may be due to the complexity of DTR and mDTR surgeries, as well as the presence of multiple anastomoses. Being the first prospective, randomized and controlled study, the KLASS – 05 trial can be a guide for the applicability of DTR. Compared with the LTG – as mentioned above—no significant difference was obtained between the two groups in terms of postoperative complications and laboratory values. DTR does not exhibit a significant difference with LTG in terms of reflux complaints either. We are looking forward to the long-term findings of the study (37).

## Discussion

Although the global consideration of PGs is constantly changing, the increasing incidence of upper third gastric tumors makes it necessary to rethink conventional surgical approaches and to fit PGs into the prevailing therapeutic algorithm. By observing the appropriate indication criteria in terms of oncological radicality, PGs seem to be appropriate in terms of the analysis of both local recurrence and lymph node dissection. By comparing certain subtypes of PG with TG, better results were found after PG if sufficient reconstruction procedures were applied. In this regard it must be emphasized that PG reconstruction procedures accompanying lower postoperative anastomosis stenosis rates provide good results in terms of the formed reflux esophagitis, and can be accepted in terms of performance and surgical difficulty. Instead of the DTR/mDTR procedure thought to be applied most frequently nowadays, the recently appeared EG modifications combining the simplicity of stapler anastomoses with antireflux mechanism (mDFT, SOFY, mSOFY) may offer a good alternative. The selection of the ideal reconstruction procedure is limited by the lack of prospective analyses, therefore, further RCTs for a wide range of patients are needed for the most optimal decision. Although the ideal reconstruction procedure after PG has still not been found, the gradual expansion of PGs is out of question. This tendency is typical mostly in Asian countries, however, the results of the studies proving the safety of PGs cannot be disregarded by surgeons in Western societies either. Our article has many limiting factors which should be noted. The majority of the presented data was provided after retrospective analysis. At the moment, no critical conclusion can be drawn without the long-term findings of high-volume prospective studies, such as the KLASS-05 trial. Nevertheless, similar to Asian countries, a paradigm shift in the care of early upper-third gastric cancer is necessary for Western countries. In this regard, the technical feasibility and oncological radicality of PG may become less of an issue with the correct indication. In terms of reconstructive procedures, the combination of stapler anastomoses with the flap technique can provide both technical and functional advantages, and may become the standard of PG surgery in the future.
